# P2Y1R and P2Y2R: potential molecular triggers in muscle regeneration

**DOI:** 10.1007/s11302-022-09885-z

**Published:** 2022-07-29

**Authors:** Min-Jia Wang, Bi-Ru Yang, Xin-Yu Jing, Yao-Zheng Wang, Lu Kang, Kai Ren, Liang Kang

**Affiliations:** 1 School of Sports Medicine and Health, Chengdu Sport University, Chengdu, 610041 China; 2 Department of Postpartum Rehabilitation, Sichuan Jinxin Women & Children Hospital, Chengdu, 610066 China; 3 Zigong First People’s Hospital, Sichuan Vocational College of Health and Rehabilitation, Zigong, 643000 China; 4 Institute of Sports Medicine and Health, Chengdu Sport University, Chengdu, 610041 China

**Keywords:** P2Y1, P2Y2, MuSCs, Muscle regeneration, ERK1/2, p38-MAPK

## Abstract

Muscle regeneration is indispensable for skeletal muscle health and daily life when injury, muscular disease, and aging occur. Among the muscle regeneration, muscle stem cells’ (MuSCs) activation, proliferation, and differentiation play a key role in muscle regeneration. Purines bind to its specific receptors during muscle development, which transmit environmental stimuli and play a crucial role of modulator of muscle regeneration. Evidences proved P2R expression during development and regeneration of skeletal muscle, both in human and mouse. In contrast to P2XR, which have been extensively investigated in skeletal muscles, the knowledge of P2YR in this tissue is less comprehensive. This review summarized muscle regeneration via P2Y1R and P2Y2R and speculated that P2Y1R and P2Y2R might be potential molecular triggers for MuSCs’ activation and proliferation via the p-ERK1/2 and PLC pathways, explored their cascade effects on skeletal muscle, and proposed P2Y1/2 receptors as potential pharmacological targets in muscle regeneration, to advance the purinergic signaling within muscle and provide promising strategies for alleviating muscular disease.

## Introduction

The proportion of skeletal muscle varies according to age, gender, race, and physical activity, accounting for 40–50% of total body mass [1, 2]. It can respond to stimuli modifying its homeostasis, such as atrophy, hypertrophy, and regeneration [3]. Trauma (sharp or blunt), ischemia, and muscular disease (such as muscular dystrophy or sarcopenia), as well as the muscle’s contraction (such as eccentric contraction), may cause skeletal muscle injury [2, 4, 5]. The skeletal muscle has the extraordinary ability to rapidly start an extensive heal process, usually in the first week after injury, and peaks during the second week after injury, and then uncontrollably declines. The activation, proliferation, differentiation, fusion of muscle stem cells (MuSCs) and the formation of new myotubes have been reported to regenerate muscle. Reportedly, many of the therapeutic modalities are for promoting muscle regeneration to treat muscle injury, including cryotherapy and thermal ultrasound, nonsteroidal anti-inflammatory drugs, and platelet-rich plasma (PRP). However, these therapeutic modalities still lack laboratory and/or clinical evidence [6]. To find a reliable application for the treatment, it is crucial to clarify the molecular mechanism and understand the clinical effects and drug treatment strategies.

It is noteworthy that purinergic signaling is involved in the process of muscle regeneration. Nucleotides released from cells due to stress, injury, or inflammation bind and activate the G protein-coupled receptors (GPCRs) on the cell surface, regulating the intracellular reaction. The GPCRs binding to the Gq/11 family on skeletal muscle transmit extracellular signals into the cell by purinergic signaling, which containing P2YR. The elevated ATP after skeletal muscle injury can directly activate P2R in skeletal muscle. P2Y2 signals through phospholipase C (PLC), Ca^2+^, ERK, and p38 MAPK to induce cell activation, proliferation, and migration. Evidence indicates the expression of multiple P2R and the indispensable function of P2Y2R in muscle development [7]. In muscle development, purines bind to its specific receptors, including P2Y1/2/4/6/12 receptors, which is responsible for regulation of C2C12 (mouse myoblast) proliferation by modulating ERK class kinase activity[8].

In addition, P2Y is also involved in skeletal muscle regeneration. Skeletal muscle has remarkable regeneration capabilities, mainly due to its resident MuSCs, which proliferate, differentiate into fusion-competent myoblasts, and facilitate muscle regeneration, activation, and muscle repairing, regulated by a variety of factors such as hepatocyte growth factor (HGF), basic fibroblast growth factor (bFGF), insulin-like growth factor-1 (IGF-1), nerve growth factor (NGF) which have been confirmed and reported. Additionally, existing evidence showed that P2Y1 and P2Y2R had been identified in human skeletal muscle at the transcriptional level. P2Y1/2R may participate in the muscle regeneration process by regulating MuSCs with purinergic signals and molecules. Current clinical drug research on P2Y1/2R targets is focused on cardiac and smooth muscle-related diseases. Clinical studies show that human c-Kit + cardiac progenitor cells (hCPCs) are a promising therapeutic approach for the treatment of heart failure. The hCPCs’ proliferation and migration significantly improved by overexpressing or stimulating P2Y2R [9]. In addition, in vitro research found that P2Y1R activation induces apoptosis in PCa cells through the Capase3/7 and ROS signaling pathways [10]. The signaling pathway involves opening small conductance calcium-activated potassium channels (K^+^-Ca^2+^ family) that result in smooth muscle hyperpolarization and relaxation [11]. Thus, P2Y1R plays an essential role in inhibitory neuromuscular transmission in the gastrointestinal tract. Although the existing evidence indicated that P2Y1R or P2Y2R involves in skeletal muscle regeneration, the clinical application of P2Y1R or P2Y2R in muscular disease hasn’t been reported. In this review, we sort out the relevant purinergic signal pathways that might be related to muscle regeneration, explore their effects on skeletal muscle, clarify the molecular mechanism of muscle regeneration, and propose the hypothesis based on the potential impacts of P2Y1/2 and provide a promising target for the muscular disease.

## Muscle regeneration and purine receptors on muscle

The initial phase of muscle regeneration is characterized by necrosis of the damaged tissue and activation of an inflammatory response. This phase is rapidly followed by activation of myogenic cells to proliferate, differentiate, and fuse leading to new myofiber formation and reconstitution of a functional contractile apparatus. Activation of MuSCs is a crucial element in this process [12]. MuSCs, the primary cell type involved in skeletal muscle regeneration [13], are heterogeneous and exist between the sarcolemma and the basal lamina of muscle fibers [14, 15]. In mature resting muscles, MuSCs are predominantly quiescent and be activated and re-enter the cell cycle by proliferating and differentiating into myoblasts and repairing the injured area after injury or degeneration[16, 17].

**Fig. 1:**
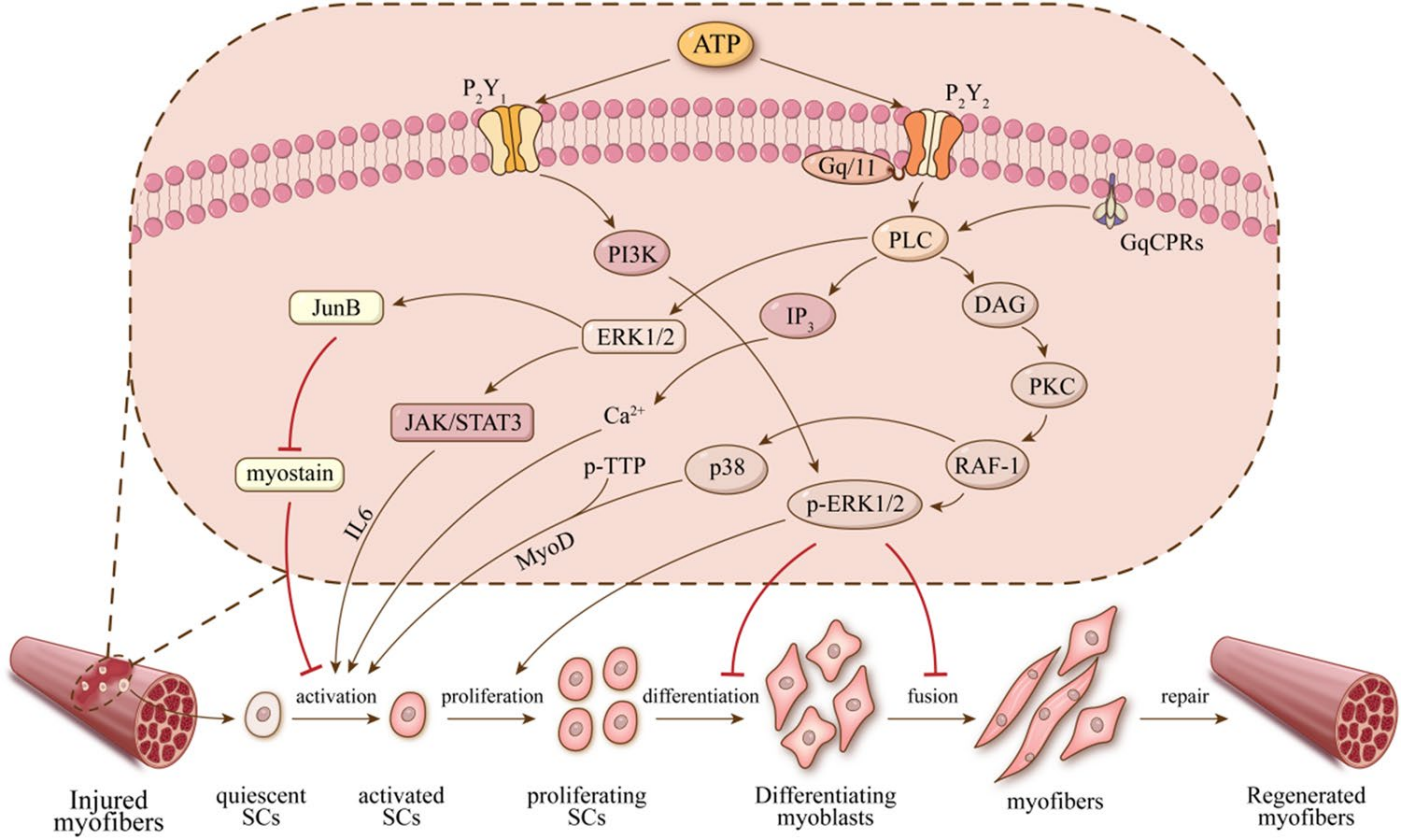
ATP participates in muscle regeneration through P2Y1/2R purinergic signaling

Purines binding to specific receptors during muscle regeneration, such as P2Y1/2/4/6/12 and P2X4/5/7 receptors, regulate C2C12 proliferation by modulating ERK class kinase activity [18]. The eATP is considered a co-transmitter released by motor nerves and can also be released from stress removed under nonlytic stimuli conditions such as hypoxia, inflammation, or cell swelling [19]. In the muscle, ATP is primarily known for its function as an energy source and as a mediator of the “excitation–transcription” process, which guarantees muscle plasticity in response to environmental stimuli [20]. Extracellular ATP through P2R induced inositol phosphate accumulation before and after myoblast fusion. This result may provide the possibility that ATP participates in muscle production through P2R by inositol phosphate accumulation [21] (Fig. 1).

Evidence proved that P2R is expressed on skeletal muscle and that the expression pattern varies with the developmental stage and species. Sequential expression of P2X2, P2X5, and P2X6 receptors has been described for developing rat skeletal muscle [22], while P2X1, P2X4, P2X5, and P2X6 receptors have been demonstrated in developing chicken myoblasts [23–25]. In human skeletal muscle, low levels of P2X7R have been detected [26]. ATP regulates mammalian skeletal muscle differentiation by activating P2X5R on satellite cells [27]. P2XR plays essential roles in the early development of skeletal muscle. However, the expression levels of P2XR are immediately reduced and often functionally undetectable after birth [8, 24]. In addition, P2X2, P2X5, and P2Y1 were found strongly expressed in the adult mdx mouse model (as a surrogate for Duchenne muscular dystrophy (DMD) research) muscle; the P2X5 and P2Y1 receptors were expressed first on activated satellite cells, P2Y1R also on a range of immune cells. Since some cytokines, including IL-6 and LIF, can act as growth factors in damaged skeletal muscle, it is possible that P2Y1R expression may be involved in regulating leukocyte activity and indirectly muscle repair [28]. This was followed by the expression of the P2X2R on newly formed myotubes [29]. It is the first evidence of a role for purinergic signaling in muscle regeneration in vivo. It indicates that P2X5, P2Y1, and P2X2 receptors play positive roles in MuSCs’ activation and myotube formation, respectively. These findings strongly suggest that purine receptors participate in the specific processes of muscle regeneration, including MuSCs’ activation, proliferation, differentiation, fusion, and new myotube formation in various forms. In rat embryonic skeletal muscle cells, similar early expression of the P2Y1R has been found. In contrast, the expression of the P2Y2R gradually increased with development, and the presentation of the P2Y4R was also high in early and late embryos. In contrast, the skeletal muscles of rats aged 3 weeks or 2 months showed that the expression of the P2Y4R was downregulated, and the expressions of P2Y1 and P2Y2 receptors were only detected in small cell groups between muscle fibers, which were preliminarily identified as satellite cells. This result indicates that P2Y1 and P2Y2 receptors on MuSCs may be the potential target of muscle regeneration, and more research is required in the future [30]. Nevertheless, the current study found the specific role of P2Y2R on differentiated skeletal muscle cells. An indispensable event for muscle regeneration is cell proliferation, and existing evidence shows that P2Y1 and P2Y2 receptors have been identified in human skeletal muscle at the transcriptional level [31]; P2Y2R might play a role in activation, signaling, and regulatory molecules during proliferation [2]. It was found that the P2Y2R regulates MuSCs via phosphorylation of ERK1/2 and P38 MAPK pathways.

## The GPCRs binding to the Gq/11 family on the skeletal muscle transmit extracellular signals into the cell

Hormones, neurotransmitters, neuropeptides, chemokines, autocrine, and paracrine signals transmit their functions to target cells through the interaction between G protein-coupled receptors (GPCRs) and heterotrimer G protein [32]. A group of rhodopsin-like GPCRs are responsible for many essential functions in physiology and pathology, such as platelet aggregation, immune responses, neuroprotective effects, inflammation, and cellular proliferation [33]. Each of the four major subfamilies of G proteins is associated with different signaling pathways: Gq/11 activates the PLC family, Gs stimulates the adenylyl cyclase (AC) pathway, Gi/o inhibits, AC and G12/13 activate small GTPases [34].

Through Gαq-mediated activation of phospholipase C(PLC), activation of P2Y2R induces the production of inositol 1,4,5-trisphosphate(IP3), thereby causing Ca^2+^ to be released from the sarcoplasmic reticulum (SR)-localized inositol 1,4,5-triphosphate receptors (IP3R), Gq-related PLC-β and PKC/Ca^2+^ pathways. PLC-β is the most well-known downstream effector molecule of GqPCR. The canonical pathway for the Gq/11 family is the activation of PLC-βenzymes, which catalyze the hydrolysis of the minor membrane phospholipid phosphatidylinositol bisphosphate (PIP2) to release IP3 and DAG. The Gq/11 subfamily, including Gq, G11, G14, and G15/16, shares structural similarity, and activation of the subunit within each protein complex can activate PLC-β [34, 35]. Furthermore, all of these four subunits regulate both overlapping and distinct signaling pathways, thereby stimulating inositol lipid (i.e., PKC/calcium) signaling through PLC-β isoforms [32, 36, 37].

These second messengers propagate and amplify the GqPCR-mediated signal with calcium mobilization following release from IP3-regulated intracellular stores and DAG-mediated stimulation of PKC activity. Inositol lipids, DAG, PKC, and calcium participate in multiple signaling networks, linking Gq family members through various cellular events. The study indicates that ATP-induced activation of the P2Y receptor and the subsequent increase in [Ca^2+^] i through IP3R convert a mechanical load into activation of intracellular signaling pathways such as phosphatidylinositol 3-kinase (PI3K)/Akt, subsequently leading to muscle hypertrophy [38].

## P2Y1R inhibits skeletal muscle differentiation and fusion by activating PI3K/ERK pathway in a Ca^2+^-independent manner

P2Y2R are G protein-coupled receptors, which mainly activate phospholipase C, leading to the formation of inositol IP3 and mobilization of intracellular calcium ([Ca^2+^]i) [39, 40]. On the contrary, ATP inhibits Ins(1,4,5) P3-evoked Ca^2+^ release in smooth muscle via P2Y1Rs which does not require PLC[41]. Studies have shown that ATP depends on PLC-mediated PI3K activity to activate ERK1/2. However, the relationship between the P2Y1R and PLC and PI3K is unclear [42]. In addition, the research found the PI3K inhibitor Ly294002 decreased the ERK1/2 phosphorylation by ATP significantly, which verifies that result [43].

A study using quiescent muscle stem cells as a paradigm of cell activation following injury, capturing early cell activation following muscle injury, found that an essential ERK1/2 primary proliferation signal precedes the initiation of the Notch-regulated myogenic program. It demonstrated that an early ERK signal is the driving force of MuSCs proliferation; ERK signal maintains the capacity of these cells to enter the activation phase [44]. It has been found that denervation or squeezing of a motor nerve in chicken or rat leads to a reduction of P2Y1R transcript in muscle by up to 90%, which is recovered during regeneration. Further, the activation of the P2Y1R by adenine guanosine monophosphate stimulated the accumulation of inosinic acid in muscular tubes and the mobilization of intracellular Ca^2+^ in cultured chick myotubes [45].

Early research has shown P2Y1Rs seem to be stimulated by endogenous ATP; this happens in early damage [46]. Then a study confirmed ATP could trigger P2Y1Rs. After muscle injury, mdx mice model (the most commonly used DMD model) muscle may contain a high level of extracellular ATP, and it is known that ATP and other extracellular nucleotides can affect the activity of MuSCs. Therefore, a study used an mdx mouse model to study the expression of P2 receptors in regenerated muscle in vivo and in vitro and found that P2Y1Rs were expressed in activated MuSCs and infiltrating immune cells. It primarily provides evidence of the role of P2Y1 in muscle regeneration in vivo, indicating that purinergic receptors may have therapeutic effects on degenerative muscular disease. P2Y1R was strongly expressed in adult mdx muscle, first activated MuSCs and a range of immune cells. These findings strongly suggest a role for purinergic signaling in the process of skeletal muscle regeneration. Immunolabeling of sequential muscle sections demonstrated that the marked increase in immunoreactivity was due to the expression of the P2Y1R on a subpopulation of activated MuSCs (as identified by the expression of the myogenic transcription factors MyoD or myogenin). The absence of significant immunoreactivity for either of these receptors in mononucleated cells in control muscle samples suggested that purinoceptors were not expressed by quiescent MuSCs but only activated MuSCs [47].

## P2Y2R promotes skeletal muscle regeneration and acts as the switch of [Ca^2+^]i-dependent activation of PCL pathways

P2Y2Rs regulate the activation, proliferation, and differentiation of MuSCs through the PLC pathway to repair skeletal muscle. ATP is believed to bind to a G protein-coupled P2YR (e.g., P2Y2R), contribute to the activation of PLC, and promote phosphatidylinositol hydrolysis, which generates diacylglycerol (DAG) and inositol IP3, and stimulate PKC and cytosolic calcium ([Ca^**2+**^]i) mobilization [38, 48, 49]*.*

P2Y2R stimulates the activation of resting MuSCs by the PLC pathway. The ERK1/2 has a role in MuSCs’ activation, which is a subtype of the mitogen-activated protein kinase implicated in skeletal muscle growth and differentiation regulation. It was proved that JunB and interleukin-6 (IL-6) are the potential downstream targets of ERK1/2. Evidence indicated that the transcription factor JunB is also a significant determinant of whether adult muscles grow or atrophy [50]. The transcription of JunB and IL-6 is upregulated by activating P2Y2R through [Ca^2+^]i-dependent activation of ERK1/2 and p38 MAPK pathways, respectively. ATP acts as the regulator of skeletal muscle hypertrophy, and P2Y2R acts as the switch [38, 51]. JunB transcription factor is essential for maintaining muscle size, inducing rapid hypertrophy, and blocking atrophy. Interestingly, unlike many other transcription factors related to muscle differentiation and muscle atrophy, JunB does not affect satellite cell proliferation and stimulates protein synthesis independent of the Akt/mTOR pathway [50]. In previous studies, it has been found that FoxO3 expression induces Smad3 phosphorylation and the activation of atrogin-1 and MuRF-1 promoters. On the contrary, JunB overexpression leads to the dephosphorylation of Smad3, which can inhibit the expression of myostatin, thus reducing protein decomposition, which may contribute to JunB-induced muscle growth [50, 52]. In addition, IL-6 is well-known as a pleiotropic cytokine associated with the control and coordination of immune responses [50] and an essential regulator of hypertrophy muscle growth mediated by MuSCs. The lack of IL-6 can eliminate the satellite cell proliferation and muscle nucleus proliferation in the pre-existing muscle fibers by damaging the activation of STAT3 and the expression of its target gene cyclin D1, thus reducing muscle hypertrophy in vivo [53]. IL-6 has concentration- and time-dependent effects on C2C12 myoblasts and primary human myoblasts. Low IL-6 concentration induces proliferation, while high IL-6 concentration induces differentiation. These effects are mediated by specific JAK/STAT/SOCS pathway components. It was proposed that different JAK/STAT pathway combinations have opposite effects on muscle differentiation and myogenesis. Indeed, the JAK1/STAT1/STAT3 axis promotes myoblast proliferation, preventing premature differentiation into myotubes. Conversely, JAK2/STAT2/STAT3 induces myogenic differentiation, suggesting that other intracellular ligands act on JAK/STAT factors to obtain distinct cellular responses at each step during development and myogenesis [54]*.* STAT3 induces transcription of downstream genes involved in several biological functions, including proliferation, differentiation, and survival of myoblasts. Studies have found that the muscle cells of IL-6 KO mice show a decrease in proliferation ability in vivo and in vitro. This damage is caused by the lack of activation of the STAT3 signal mediated by interleukin-6, which proves the role of the JAK/ STAT pathway in regulating the myogenic progress of adult MuSCs [55]. STAT3 is a critical signaling protein engaged by the exogenous factors that drive these processes. Interestingly, the activation or loss of STAT3 can lead to insulin resistance, the loss of muscle mass, or the increased repair of MuSCs depending on the stimuli and the duration of STAT3 depletion. IL-6 drives insulin resistance in cultured skeletal myotubes derived from people with impaired glucose tolerance. Some data would suggest that inhibiting STAT3 in muscle will increase insulin sensitivity and facilitate muscle repair through promoting satellite cell expansion and repair in human dystrophic tissue [53, 56]. Under acute conditions (such as acute resistance exercise and resistance training), the increase of muscle cell proliferation mediated by the IL-6/STAT1/STAT3 pathway leads to muscle hypertrophy, suggesting the potential role of STAT3 in muscle cell-mediated adaptive growth of skeletal muscle [57, 58]. Significant, sudden, and acute induction of the IL-6 cascade promotes muscle growth. At the same time, IL-6 sustained and elevated release and STAT3 activation have been associated with muscle atrophy in several catabolic conditions, such as obesity, diabetes, and age-induced sarcopenia or cancer [59]*.* Overexpression of IL-6 in transgenic mice resulted in muscle atrophy, although this situation was completely reversed by treatment with membrane IL-6 receptor antibody. In in vitro and in vivo experiments, neutralizing antibody treatment prevented the increase of IL-6 concentration, protected the weight loss of cachexia mice, and blocked STAT3 activation to reduce muscle wasting [59–63].

IP3 produced by the activation of PLC can also activate the quiescent MuSCs. Studies have pointed out that Ca^2**+**^ plays an essential role in the activation of ERK1/2, which is reflected in the intracellular and extracellular, respectively. The removal of extracellular Ca^2**+**^ strongly inhibits the activation of ERK1/2 nucleotides. In addition, ATP induces the mobilization of intracellular Ca^**2+**^, which is due to the direct effect of IP3 on endoplasmic reticulum storage [18]. DAG downstream of PLC activates PKC, causing cell reaction, including p38 MAPK. By investigating the expression patterns of PKCθ (expressed in skeletal muscle) in normal and regenerating tibialis anterior muscles in rats, it has been found that PKCθ protein was identified in quiescent MuSCs and half the differentiating MuSCs labeling with myogenin. It was not observed in proliferating MuSCs by marking with bromodeoxyuridine (BrdU) in the regenerating muscle. This indicated that PKCθ might be essential in inhibiting differentiation and maintaining the quiescent MuSCs in muscle regeneration [64].

The p38 MAPK signaling, a subgroup of the MAPKs, was first described as a transducer of the stress conditions and a critical mediator of inflammatory cytokines. Still, many different non-stress stimuli can also activate the p38 MAPK pathway, regulating multiple cellular processes or differentiation of various cells [65]. Treatment with the p38α/β inhibitor SB203580 prevented the fusion of myoblasts into myotubes and the induction of muscle-specific genes [66, 67]. Notably, a report has shown the requirement for p38α/β to activate the quiescent satellite cell, although the mechanism underlying this effect remains unknown [68]. Some in vitro studies showed p38 MAPK regulates myogenesis through a distinct mechanism. It is found that p38 is rapidly activated in myocytes induced by cell differentiation, and this activation is maintained during myotube formation. It differs from another MAPK pathway, Jun-N-terminal kinase (JNK) stimulation, triggered by stress and cytokines [67]. Besides, independent findings published over the past few years have shown that the p38 MAPK pathway plays a key role in the control of muscle gene expression at different stages of the myogenic process, including skeletal muscle differentiation and fusion, which is modulated by the sequential activation of MRFs and their transcriptional coactivators, including chromatin remodeling enzymes [69–72]. The p38 MAPK signaling induces the activation of quiescent MuSCs and MyoD induction [68]. The study identified p38α/β MAPK-mediated phosphorylation of TTP regulates MyoD mRNA decay, thereby regulating MuSCs’ activation [73]. However, research suggests that aberrant p38 MAPK signaling can inhibit MuSCs’ regenerative capacity; this result is in contrast to previous studies [74].

In contrast, a study has been found that P2Y2-mediated ERK1/2 phosphorylation through the PLC pathway may inhibit the differentiation into myoblasts and fuse into myofibers, resulting in muscle atrophy. In proliferating cells, RAS-induced ERK signaling primarily controls the G1/S-phase transition of the cell cycle [75]. The study proved that the Ras-ERK pathway plays a critical role in the inhibition of myocyte differentiation and muscle regeneration, which leads to muscle atrophy by inhibiting differentiation into myoblasts and fusing into myofibers [76].

## Conclusion

P2Y1R and P2Y2R play molecular roles in skeletal muscle regeneration during or after injury, aging, and disease. In this pathological condition, ATP is elevated, activating the P2Y1R and P2Y2R. P2Y1R triggers the phosphorylation of ERK1/2 to promote MuSCs’ proliferation, inhibit differentiation into myoblasts, and fuse into myofibers, relying on the PI3K activity but is independent of the Ca^2+^ signal. Meanwhile, P2Y2R participates in muscle repair and regeneration, which depends on PLC pathways and activates downstream reactions to stimulate the activation of resting muscles. Among them, JAK/STAT3 is stimulated by the ERK1/2 pathway, Ca^2+^ is increased by IP3, and MyoD is decreased by the PKC pathway, which depends on the phosphorylation of TTP. In addition, P2Y2R mediated ERK1/2 phosphorylation through the PLC pathway and stimulated the proliferation of MuSCs. In this review, we summarized muscle regeneration via P2Y1R and P2Y2R and speculate that P2Y1R and P2Y2R might be potential molecular triggers for MuSCs’ activation and proliferation via the p-ERK1/2 and PLC pathways and provide a reference for the positive molecular mechanism in muscle regeneration.

Recent studies of P2YR in skeletal muscle are mainly conducted in vitro and in animal experiments, such as mice and human muscle cells. The molecular mechanism of P2YR in skeletal muscle and its clinical practice will be one of the research directions in the future. Furthermore, the clinical application of P2Y1R or P2Y2R in muscular disease hasn’t been reported. Based on the existing evidence of P2Y1R and P2Y2R involved in skeletal muscle regeneration, the specific antagonist and agonist of P2Y1R or P2Y2R as promising candidates for skeletal muscle repair are worth exploring.

## Data Availability

Not applicable.
